# Seroprevalence of border disease virus and other pestiviruses in sheep in Algeria and associated risk factors

**DOI:** 10.1186/s12917-018-1666-y

**Published:** 2018-11-12

**Authors:** Naouel Feknous, Jean-Baptiste Hanon, Marylène Tignon, Hamza Khaled, Abdallah Bouyoucef, Brigitte Cay

**Affiliations:** 1 LBRA, Institute of Veterinary Sciences, Saad Dahlab University, Soumaa Road, BP 270, 09000 Blida, Algeria; 2 Sciensano, Infectious animal diseases directorate, Service of enzootic, vector-borne and bee diseases, Groeselenberg 99, 1180 Brussels, Belgium; 3 ENSV, National superior veterinary school, Bab ezzouar, El allia Algeria

**Keywords:** Border disease virus, Pestivirus, Seroprevalence, Sheep, Algeria, Persistently infected

## Abstract

**Background:**

Border disease virus (BDV) is a pestivirus responsible for significant economic losses in sheep industry. The present study was conducted between 2015 and 2016 to determine the flock seroprevalence of the disease in Algeria and to identify associated risk factors. 56 flocks from nine departments were visited and 689 blood samples were collected from adult sheep between 6 and 24 months of age (*n* = 576) and from lambs younger than 6 months (*n* = 113). All samples were tested by RT-PCR as well as by Ag-ELISA, to detect Persistently Infected (PI) animals. Serum samples from adults were tested by Ab-ELISA (Enzyme Linked Immuno-Sorbent Assay), to detect specific antibodies against pestivirus and 197 of them were further characterized by VNT (virus neutralization test) for the detection of neutralizing antibodies specific for BDV and for Bovine virus diarrhea virus (BVDV-1 and BVDV-2).

**Results:**

No PI animals were found among the 689 sheep tested. 144/197 sera were positive in VNT for BDV, and 2 sera were strongly positive BVDV-2. Fifty-five flocks (98%) had at least one seropositive animal and the apparent within-flock seroprevalence was estimated to be 60.17% (95% C.I.: 52.96–66.96). The true seroprevalence based on estimated sensitivity and specificity of the Ab-ELISA was 68.20% (95% C.I.; 60.2–76.3). Several risk factors were identified as linked to BDV such as climate, landscape, flock management and presence of other ruminant species in the farm.

**Conclusion:**

These high seroprevalence rates suggest that BDV is widespread and is probably endemic all over the country. Further studies are needed to detect and isolate the virus strains circulating in the country and understand the distribution and impact of pestiviruses in the Algerian livestock.

**Electronic supplementary material:**

The online version of this article (10.1186/s12917-018-1666-y) contains supplementary material, which is available to authorized users.

## Background

In accordance with the ICTV (International Committee on Taxonomy of Viruses), BDV (Border Disease Virus) belongs to the *Flaviviridae* family which includes four genera: *Flavivirus*, *Hepacivirus*, *Pegivirus,* and *Pestivirus*; the latter previously included four species: bovine viral diarrhea virus 1, (BVDV1), bovine viral diarrhea virus 2 (BVDV2), classical swine fever virus (CSFV), D and border disease virus (BDV). Since 2017, these four species have been renamed *Pestivirus A, B, C, and D,* respectively*.* Seven other species have been added in the genus, namely *Pestivirus E* to *K*, including giraffe pestivirus (*Pestivirus G),* Hobi-like pestivirus (*Pestivirus* H) and other atypical species isolated in wild and domestic mammals. The 11 currently recognized pestivirus species are now named in relation to molecular and antigenic relatedness in a host-independent scheme [1]. BVDV can infect cattle, sheep, goats, pigs and other ungulate species [2] and infection of sheep by BVDV-1 and BVDV-2 in natural and experimental conditions was demonstrated [3]; in some regions BVDV prevalence in sheep can be higher than BDV [4]. CSFV seems to be restricted to pigs and wild boars [5]. Although BDV is generally considered as an agent for a sheep disease, it is not strictly host specific and can cross infect cattle, sheep, goats, pigs and non-domesticated species [6]; transmission of BDV between small ruminants and cattle has been described by several authors [7–9]. BDV infection can cause significant economic losses to sheep industry due to its impact on reproduction and health. Clinical signs in sheep are dominated by infertility, abortions, stillbirths, or even the birth of lambs with hairy fleeces called “hairy-shaker” or “blurred” or an abnormal body conformation. BDV can also cause a condition similar to mucosal disease [10]. The main source of infection in a flock are the PI sheep, which are born infected and spread the virus during their whole life. PI lambs result from transplacental infection of the fetus before the 60th day up to the 80th day of gestation, when the immunological system is still immature [6]. Border disease is present in several continents and seroprevalence rates in sheep range from 5 to 50% depending on the country or the regions within a same country [11]. However, prevalence of BDV in Algeria, where vaccination is not practiced remains completely unknown and there has been no scientific publication on the topic so far to our knowledge. The purpose of this study conducted between 2015 and 2016 was to estimate the BD seroprevalence and shedding in Algerian sheep flocks and to identify associated risk factors. Such epidemiological data should contribute to improve the visibility of this neglected disease and to develop a monitoring plan for the country.

## Results

### Flock and within-flock seroprevalence

A flock was considered positive for ruminant pestivirus when at least one animal was positive in Ab-ELISA. All flocks except one were seropositive, therefore the flock seroprevalence was estimated to be 55/56 = 98.2% (95% C.I. 90.5–99.6). The proportion of positive sheep in each flock ranged between 1 and 100%. Out the 576 sera tested, 344 samples were considered as seropositive (304 positive + 40 doubtful in Ab-ELISA). The apparent overall within-flock seroprevalence, based on the GEE model was estimated to be 60.17% (95% CI: 52.96–66.96). The true overall seroprevalence, taking into account our estimation of Se (84.0%) and Sp (92.4%) of the Ab-ELISA (see below) was calculated to be 68.20% (95% C.I. 60.2–76.3).

The within-flock seroprevalence by departments is described in Table 1. There were marked regional differences in the flock prevalence, ranging from 18% in Chlef (95% C.I. 5.1–30.9) to 100% in Setif. However, our sampling design was built to estimate with a reasonable precision the overall within-flock seroprevalence but cannot provide an accurate estimation at department level (this would have required a larger number of flocks in each department). This is the reason why the 95% CI at regional level were large and the differences of within-flock prevalence between regions were not statistically significant except for the prevalence in Chlef which was significantly lower (*p* < 0.0001) than the prevalences in Djelfa, Al Bayadh, Msila, Saida and Laghouat.

**Table 1:** Ovine population (number of flocks), sampling performed and estimated (apparent) seroprevalence (with 95% CI) of Border disease, by department, according to GEE model

Department	Ovine flocks	N Fl	N Lb	N Ad	N Pos	Prev %	95% CI
El Bordj	9000	3	0	30	15	50.00	[27.33; 72.67]
Setif	4230	1	0	10	10	100.00	n.d.
Msila	23,000	7	31	71	51	71.90	[60.36; 81.13]
Djelfa	21,000	6	5	60	35	58.33	[43.12; 72.11]
Laghouat	23,000	8	24	80	39	51.07	[36.55; 65.41]
El Bayadh	25,000	7	0	70	57	81.43	[64.40; 91.40]
Tizi Ouzou	18,000	5	1	50	23	46.00	[20.87; 73.34]
Chlef	17,230	5	30	50	9	18.00	[09.90; 30.50]
Saida	50,000	15	22	155	105	67.68	[55.81; 77.64]
Total	190,460	57	113	576	344	60.17	[52.96; 66.96]

N Fl: Number of sampled flocks

N Lb: Number of sampled lambs (animals < 6 months, plasma)

N Ad: number of sampled adults (serum + plasma)

N Pos: number of positive sera (adults)

Prev %: within-herd seroprevalence

### Comparison between ab-ELISA and VNT results

The number of positive, doubtful and negative samples among the 576 sera tested by ELISA-Ab was 304, 40, and 232 respectively. A list of all samples with their respective Ab-ELISA and VNT results is provided in Additional file 1: Table S1.

To estimate the performances (Se and Sp) of the pestivirus Ab-ELISA compared to VNT, 197 sera were tested in parallel by Ab-ELISA and by three different VNT (BDV, BVDV-1 and BVDV-2). Table 2 gives the number of positive, doubtful and negative samples when tested by Ab-ELISA compared to the BDV-VNT titer. Table 3 gives the number of positive, doubtful and negative samples in Ab-ELISA compared to the ratio [VNT titer for BDV / VNT titer for BVDV], the latter being calculated compared to the highest titer in BVDV, whether it was BVDV-1 or BVDV-2. This ratio was split into three categories: (a) BDV titer = four folds the BVDV-1 or BVDV-2 titer, (b) BDV titer = two to three folds the BVDV-1 or BVDV-2 titer, (c) BDV titer = less than two folds the BVDV-1 or BVDV-2 titer. Samples from category (a) were considered as specifically positive for BDV.

**Table 2:** Number of positive, doubtful and negative samples in Ab ELISA, according to the manufacturer’s recommended cut-off, compared to VNT titer for BDV, considering 1/8 titer as cut-off for VNT

ELISA result	Neg VNT	Pos VNT (titer ≥1/8)												
	< 1/8	1/8	1/12	1/16	1/24	1/32	1/48	1/64	1/96	1/128	1/192	1/256	*Total Pos (VNT)*	Total samples
Doubt	4		1			7	1	5	3	10	5		*32*	36
Pos	0	1		1	1	9	5	20	3	15	6	28	*89*	89
Neg	49	2		2		7	2	5	1	2		2	*23*	72
Total	53	3	1	3	1	23	8	30	7	27	11	30	*144*	197

Neg: negative

Pos: positive

Doubt: doubtful

**Table 3:** Number of positive, doubtful and negative samples in Ab ELISA compared to the ratio [VNT titer for BDV / VNT titer for BVDV-1 or BVDV-2]

	VNT Titre BDV/ Titre BVDV				Total
	> 4 x	2–3 x	< 2 x	Neg	
ELISA Ab results					
Doubt	19	7	6^a^	4	36
Pos	63	8	18	–	89
Neg	21	–	2^a^	49	72
Total	103	15	26	53	197

> 4 x / 2–3 x / < 2 x: VNT titer for BDV compared to VNT titer for BVDV-1 or BVDV-2 more than fourfold higher / between two and threefold higher / less than twofold higher

Pos: positive

Neg: negative

Doubt: doubtful

^a^including 1 sample with high BVDV-2 Titer

Among the 197 sera tested in parallel by Ab-ELISA and VNT, 144 were positive in VNT for BDV (titer ≥1/8) (Table 2) including 103 sera with a titer four folds higher for BDV than for BVDV-1 or BVDV-2 (Table 3). Of these BDV VNT positive samples, 89 were positive, 32 doubtful and 23 negative in Ab-ELISA (Table 3). Out of the 36 sera with a doubtful result in Ab-ELISA, 32 were positive in VNT for BDV (Table 2) and most of these (31/32) had a high VNT titer (1/32 up to 1/192) (Table 2) including 19 samples with a BDV titer four folds higher than for BVDV-1 or BVDV-2 (Table 3). Based on this observation we considered the sera with a doubtful result in Ab-ELISA as seropositive samples for the rest of our analysis. Therefore, the relative sensitivity of the Ab-ELISA compared to the BDV-VNT was estimated to be [(89 positive ELISA + 32 doubtful ELISA)/ 144 positive VNT] = 84.0% and the relative specificity to be [49 negative ELISA/ 53 negative VNT] = 92.4% (Table 2). If only samples with a BDV titer four folds higher than the BDV titer, then the sensitivity of the Ab-ELISA compared to BD VNT = [(63 positive ELISA + 19 doubtful ELISA) / 118 positive BDV-VNT] = 69.5.3% (Table 3). Some positive sera with the BDV-VNT cross-reacted with BVDV-1 and BVDV-2 VNT but the titers observed for BVDV were generally low except for two samples from two different flocks which had very high titers for BVDV-2 (titer = 1/480 and 1/640 respectively).

The agreement (Cohen’s kappa coefficient) between Ab-ELISA and BDV-VNT test was 0.68 (95% C.I. 0.58–0.79).

### Seroprevalence and risk factors

The differences in the proportion of seropositive animals were not statistically significant for the following studied variables: flock size, sheep breed, presence of cattle in the farm, purchase of breeding females, purchase of sheep for fattening, abortion history, sharing breeding rams, and vaccination for sheep pox and brucellosis and other diseases. On the contrary, a significant difference in seroprevalence was found for the following variables: climate (arid versus Mediterranean; OR = 4.04), landscape (mountain versus plateau; OR = 0.49), flock management (sedentary versus transhumant; OR = 0.59), presence of goat versus no goat (OR = 0.58), other clinical diseases (OR = 0.66). The detailed results including odds ratios for these risk factors are presented in Table 4.

**Table 4:** Risk factors for being seropositive for Border Disease with corresponding number and proportion of positive samples (apparent seroprevalence), number of negative samples and associated Odds-ratio (with 95% CI)

	Positive samples (%)	Negative samples	*p* value	OR	95% CI
Climate					
arid	312 (65.5%)	164	0.0001	4.04 1	2.55–6.39
mediterranean	32 (32.0%)	68			
Landscape					
mountain	31 (44.3%)	39	0.005	0.49 1	0.29–0.80
plateau	313 (61.9%)	193			
Flock management					
sedentary	126 (52.5%)	114	0.0001	0.59 1	0.42–0.83
transhumant	218 (64.9%)	118			
Herd Composition					
mixed (goat or cattle)^a^	243 (59.1%)	168	0.644^a^	0.92^a^ 1	0.63-1.33
sheep only	101 (61.2%)	64			
sheep with cattle^a^	206 (60.6%)	134	0.611^a^	1.09^a^ 1	0.78-1.53
no cattle	138 (58.5%)	98			
sheep with goat	143 (52.8%)	128	0.001	0.58 1	0.41–0.81
no goat	201 (65.9%)	104			
Clinical diseases					
yes	126 (59.0%)	108	0.017	0.66 1	0.47–0.93
no	218 (63.7%)	124			

^a^Non significant

### Ag-ELISA and RT-PCR

All 689 individual samples were tested negative by Ag-ELISA and these negative results were confirmed by the fact that all pools of plasma samples were tested negative by RT-PCR.

## Discussion

### Seroprevalence study

After infection with a ruminant pestivirus, the detection of antibodies against the highly conserved pestivirus-NS2–3 (p80) protein by competitive ELISA provides reliable results to confirm seroconversion. Such assays have been used in different countries to conduct seroprevalence surveys for pestivirus in small ruminants [4, 12, 13]. Our results indicate an estimated flock prevalence of 98.20% and an apparent within-flock prevalence of 60.17%. The true overall prevalence was estimated to be 68.20%. In Tunisia, similar results are reported, with 95% or 52/55 of positive flocks and an animal seroprevalence of 54% ± 4% [14]. Such high levels of prevalence were also found in France, where a recent study revealed that 38 sheep flocks tested in Ab-ELISA were positive in Border disease and individual seroprevalence reached 76.5% (95% CI = 74.2–78.8%) [15]. Other serological surveys carried out in Spain, Ireland, Austria, and India revealed a high seroprevalence of ruminant pestivirus at flock level with rates varying between 58 and 70% and at the individual level between 49.3 and 83% [13, 16–18].

Several factors may be in favor of the high rates observed in Algerian flocks and thus may participate in the dissemination of the virus: keeping animals in poor housing conditions, insufficient knowledge of livestock breeders about biosecurity rules, common use of transhumance and mixing flocks of different origins, lack of periodic laboratory investigations and illegal exchanges of animals from the neighboring countries. Our results were potentially biased by the fact that random selection was performed at the municipality level and not at the flock level, as there was no available sheep flock database. The selection of the sampled flock in each municipality was done by private vets and could be considered as a convenient sampling. To minimize this bias the vets were asked to select flocks as much representative as possible of the local context.

#### Regional differences in the seroprevalence

In this survey, we observed marked regional differences in the within-flock seroprevalence of BD in sheep with estimated rates between 18 and 100% depending on the department. However, our sampling design was calibrated to estimate with a reasonable precision the overall within-flock seroprevalence but cannot provide an accurate estimation at department level (this would have required a larger number of flocks in each department). Indeed, the differences observed between departments were not statistically significant except for Chlef (*P* = 18%; 95% CI 9.90–30.50) which has a lower prevalence compared to five other departments. The lower prevalence in this department can be explained by the fact that most flocks over there are sedentary, as grazing is available throughout the year thanks to favorable environment and Mediterranean climate. Such conditions can limit the contacts with potentially infected flocks. It has been reported that the transmission of the virus depends also on the degree of contacts between animals and may be more important in animals kept in buildings with nose-to-nose contact [19] than in animals that remain in the open air. A study in Northern Ireland also reported significant regional variations in flock prevalence and attributed such differences to the levels of movement, differences between the regions in management practices, and the density of the populations of sheep in the flocks [13].

#### Virus circulation

The high seroprevalence rates observed in our study can be considered as indicative of a recent infection in some flocks given that only animals aged between 6 and 24 months were sampled. By this age, maternal antibodies have waned and the presence of antibodies is due to a recent exposure to a pestivirus [20]. Animals older than 2 years were not sampled, to exclude a bias due to the seropositivity in older animals which remain lifelong seropositive after seroconversion.

#### Vaccination

Vaccination of ruminants against pestivirus can also induce seroconversion but it is not practiced in Algeria. There is no standard vaccine for BDV, but a commercial killed whole-virus vaccine has been produced [21]. However, in Algeria there is extensive vaccination of small ruminants against sheep pox virus using a locally made vaccine prepared with cellular lineage resulting from a strain of sheep embryo. Isolation of pestivirus strains from several batches of anti- sheep pox vaccines has been reported in Tunisia [22], which could be at the origin of a wide spread of the virus in this country. According to OIE (World Organisation for Animal Health) [21], contamination of modified live virus vaccines by pestivirus have been found to be a cause of serious disease following their use in sheep (including sheep pox vaccine) and other livestock. However, in our study we did not observe a significant increase of BD seroprevalence in vaccinated flocks compared to unvaccinated ones.

### Comparison between VNT and ab-ELISA

We used a BVDV-1 and BVDV-2 cattle isolate and a BDV sheep isolate for cross neutralization study. Previous studies using a commercially available indirect ELISA (SVANOVIR BD-Ab-ELISA; Svanova Biotech), comparing Ab-ELISAs to VNT have reported a sensitivity of 94.3% and 100% and a specificity of 93.7 and 100% for sheep and goats respectively from BD virus [23, 24]. In our study, 197 samples from 20 different flocks were tested in parallel with Ab-ELISA and VNT for BVDV-1, BVDV-2 and BDV. We observed low performances of the Ab-ELISA especially for the sensitivity estimated to be 84%. A possible explanation is that the commercial ELISA we used is more adapted to European strains of pestivirus and may not detect well BDV strains circulating in Algeria, as it is well known that there is a large antigenic variability in BDV strains generally [11].

The majority of the positive samples (103/144) tested in parallel by VNT for BDV, BVD-1 and BVD-2 had a VNT titer for BDV four folds higher than the titer for BVDV-1 or BVDV-2. We can therefore conclude that the prevailing pestivirus circulating in the sheep population in Algeria is Border disease virus rather than BVDV. Surprisingly, two of the sera tested in parallel had high titer for BVDV-2. These samples came from two different farms in two separate departments (Chlef and Saida); these flocks were sedentary but share grazing with cattle. BVDV-2 is a pestivirus usually specific to cattle and rarely identified in sheep. It was initially detected in cattle of North America [25] and later in other countries. In India, a cross neutralization study on sheep and goat samples exhibited a titer more than fourfold higher to BVDV-2 in one sheep and one goat [20]. Recently, a study in Spain revealed that six of eight fetuses / lambs were positive from BVDV-2 [26]. This virus may cause abortions, and probably be highly virulent, in naturally infected sheep. However, in most cases, the primary source of BVDV in non-bovid species is unknown, although direct contact with cattle appears to be the source of initial contamination [27].

### Detection of PI animals by ag-ELISA and RT-PCR

Ag-ELISA and RT-PCR were performed in our study in order to detect PI animals but no viral antigen could be detected among the 689 samples tested, despite serological findings that showed the presence of recent infection in the flocks. There are several hypotheses to explain why we were not able to detect PI BDV among the tested sheep. First, only a limited number of animals younger than 6 months were tested in each flock (*n* = 113), which is the age category in which there is a greater chance to detect a PI. Given the low prevalence of PI sheep commonly observed and reported in previous studies, the probability to detect PI animals in a small sample size is low: in Austria, the PI prevalence was only 0.32% in sheep, and in Spain, it has been described a prevalence of 0.3; 0.6 and 0.24% [15, 28]. In addition, many lambs are slaughtered at a young age for economic purposes, decreasing the chance to detect young PI animals at the time of sampling. Finally, we did not observe typical clinical signs of BD such as nervous signs, paralysis and muscle tremor on the lambs sampled so we cannot exclude that most PI animals were dead or culled at the time of the sampling. With blood samples taken from young animals younger than six months, one could expect false negative Ag-ELISA results due to the presence of maternal antibodies derived from colostrum intake. However, these samples were tested in parallel by RT-PCR, a method which is not influenced by maternal antibodies when performed on full blood.

### Risks factors

Several significant risk factors for high BDV seroprevalence were identified in our study. Due to the limited number of flocks and animals tested and the limited study zone, these risk factors should be considered as specific to the Algerian context and generalized cautiously to other endemic countries.

The seroprevalence (P) was significantly higher in inland areas with cold and arid climate characteristics (*P* = 65.5%; OR = 4.04) than in the coastal zone with Mediterranean climate (*P* = 32.0%). A similar observation was made in a seroprevalence study in Turkey [29]. Although pestiviruses are endemic in many countries with very different climatic conditions, one cannot exclude that climatic factors such as outside temperature or hygrometry could influence the survival and dissemination of the virus in the environment (feces, fomites) and have in impact on virus transmission. In our study, a significant lower seroprevalence was observed for sheep flocks raised in the mountainous regions (*P* = 44.3%; OR = 0.49) compared to flocks raised in the plateaus (*P* = 61.9%). Our results could be explained by the fact that flocks from the coastal zone are predominantly sedentary and therefore are rarely in contact with other flocks while in inland regions, flock movements are more intense, resulting in a higher infection rate.

Indeed, a significant lower seroprevalence was observed in flocks managed in a sedentary system (*P* = 52.5%; OR = 0.59) compared to transhumant flocks (*P* = 64.9%). Transhumance is a system widely practiced in Algeria, it concerns flocks located in the steppe region, where shepherds carry their flocks in the north of the country during summer season (May to September) for more pasture and return in autumn (October) to their farms. Another movement of transhumance is observed at the beginning of winter (second half of December) a little towards the south because of the enormous temperature decreases in the steppe region.

Transhumance has already been identified as a risk factor by previous studies. The seasonal migration of flocks and the use of communal pastures for grazing make the direct or indirect exposure to other species possible, including free-living ruminants [4, 15]. In another study carried out in Syria, it has been reported that transhumant flocks, especially those travelling long distances, have a significantly higher seroprevalence with an increase of 14% compared to sedentary flocks or those moving on short distances [30].

We examined in our study the possible impact of mixing sheep with other ruminant species (cattle and/or goat). Natural cases of pestiviruses transmission from cattle to sheep and vice versa have been reported [31] and the presence of sheep is a recognized as a risk factor for the introduction of BVDV into cattle herds [32, 33] and vice-versa [4]. However, the higher seroprevalence of seropositive beef and dairy herds in Northern Ireland (> 85%) suggests that the infection pressure is more important from cattle to sheep than from sheep to cattle [34]. A Swiss serological study confirms the fact that housing sheep and cattle separately significantly reduces the seroprevalence of BVDV infection in sheep but not of BDV [35]. In our study, there was no statistically significant association between serological status at the individual level and the presence or absence of cattle on farms. On the other hand, our study revealed a significantly lower seroprevalence in flocks where sheep were mixed with goats (*P* = 52.8%; OR = 0.58) compared to flocks without goats (*P* = 65.9%). Although PI goats infected with BDV have rarely been reported [36], this does not explain the apparent protecting factor of the presence of goat observed in our study. Other confusing factors, linked to the presence of goats, could explain this observation such as flock management or environmental conditions. Surprisingly, a significant lower seroprevalence was observed in flocks where other clinical diseases were reported by the owner (diarrhea, respiratory problems, weak lambs) (*P* = 63.7%; OR = 0.66) compared to flocks without clinical signs. However, the presence of other disease in our survey was based on the declarations of the breeders and not on clinical observations leading to possible bias. Moreover, in flocks with high BDV seroprevalence the disease can be considered as endemic and not in acute phase so that the immune status of animals will make the disease circulate at low noise compared to flocks that have more naïve animals. We did not find any statistical association between the occurrence of abortion cases and the BDV seroprevalence, but again this was based on the declarations of the breeders which could be biased. In Northern Ireland it is reported that among 186 fetuses serologically tested; only one was positive for BD, concluding that pestiviruses are not an important cause of sheep abortion in in this country [13]. A similar result was drawn from a study in Tunisia [14] which concludes that despite the high prevalence of BD, it is only implicated in the abortion syndrome of a single flock out of 20 tested.

## Conclusion

This work is the first epidemiological study estimating the BDV seroprevalence in sheep flocks in Algeria. Our results provide serological evidence of widespread BDV infection in Algerian sheep population but also the presence of BVDV-2 infection. As a consequence, we can propose the following recommendations for the Algerian context: (i) a control program of pestivirus infections should be considered in sheep and other ruminant species; (ii) diagnostic tests to differentiate between BVDV-1, BVDV-2 and BDV need to be available as second line, since this can have an impact on disease control measures; (iii) locally produced live vaccine batches against other diseases should be controlled for the risk of pestivirus contamination even though vaccination did not appear as a risk factor in our study. This survey also shows the impact of some risk factors on the spread and maintenance of BDV infection, such as climate, landscape, flock management, flock composition and other concurrent diseases. On the other hand, this survey did not reveal any significant effect of the flock size, the presence of cattle, the introduction of new animals into flocks and the occurrence of abortions. Further studies aiming at the isolation of the circulating strains of Border Disease virus in Algerian sheep will help to better understand the origin and dynamics of pestivirus infections in the country.

## Methods

### Study site

**Fig. 1:**
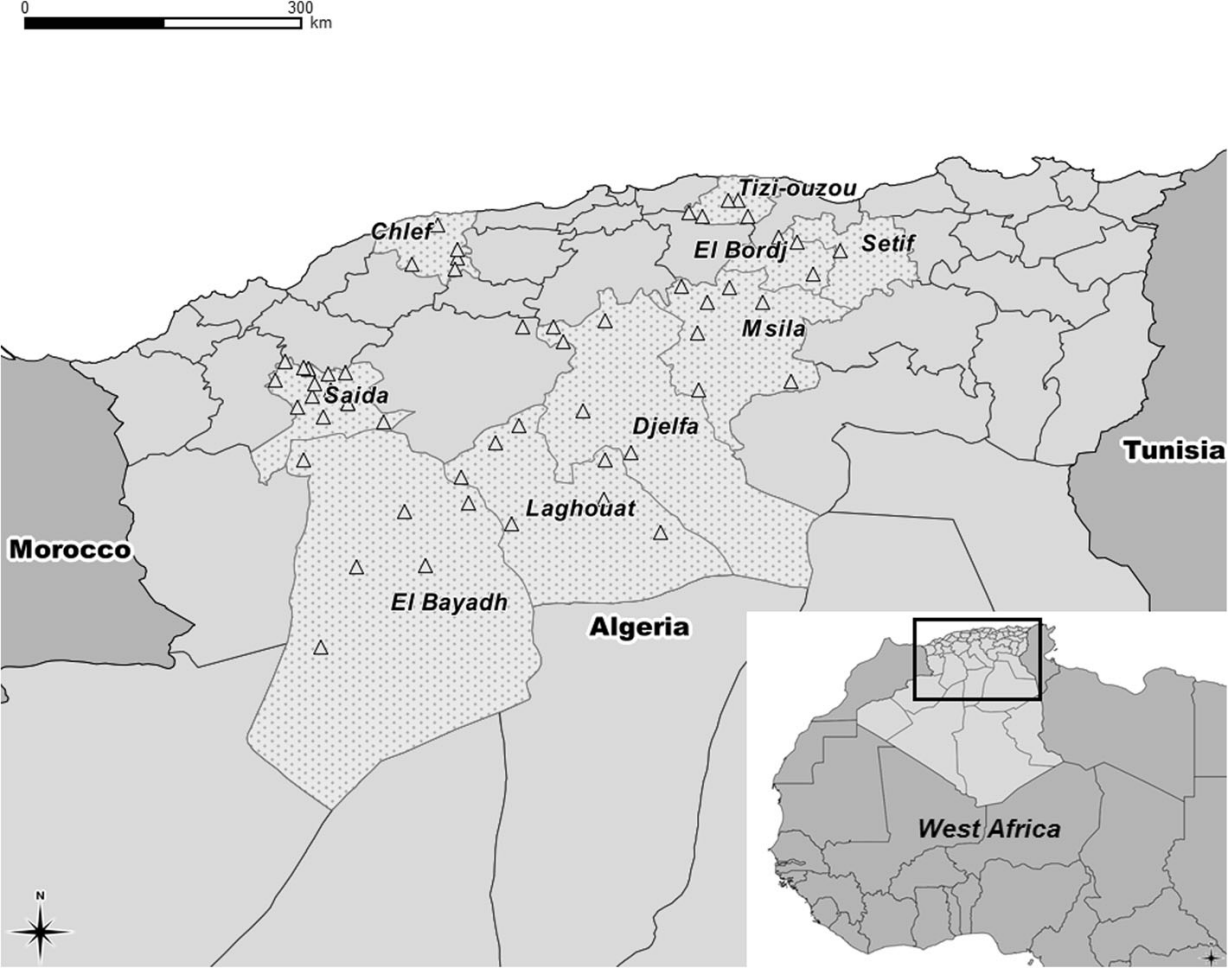
Location of the study zone (nine Algerian departments, dotted area) and of the 56 municipalities where sheep flocks were sampled (marked by Δ symbol). *(Map created using Q-GIS software and administrative maps downloaded from* *GADM.org**)*

The study was carried out in nine departments (regions) of Algeria, covering the geographical and climatic diversity of the country (Al Bayedh, Saida, Laghouat, Djelfa, Msila, El Bordj, Setif, Chlef and Tizi Ouzou). These departments were selected because of their relatively high density of small ruminants (Fig. 1). The sheep population in Algeria accounts for 80% of the total number of ruminants; it increased by more than 25%, from 21 million heads to 28 million between 2010 and 2014 [37], of which 57% is present in the area investigated. This type of sheep production is more concentrated in the steppe zone (in the north-central part of the country). During warm season, the transhumance and nomadic activities are necessary especially from May to September when pastures can no longer satisfy the food requirements of the flocks.

### Sampling

#### Sampling design

A two-stage cluster sampling was performed with a total of 576 animals (6–24 months) sampled from 56 sheep flocks originating from 56 different municipalities spread over 9 departments (Fig. 1). The sample size was calculated based on an estimated within-flock prevalence of 54% taking into account the prevalence found in a similar study in Tunisia [14] and a desired relative precision of 5%. The initial sample size (*n* = 382) was increased to 554 animals to take into account the design effect due to the cluster sampling with the following parameters: number of clusters (flocks) = 56; number of sampling units (animals) in each cluster = 10; intra-class correlation (ICC or ρ) = 0.05. We chose an ICC of 0.05 which is an intermediate value for a range of values (0.01–0.36) mentioned for BVDV in [38]. The number of flocks sampled in each department was proportionate to the number of ovine flocks estimated by department (based on data from regional agricultural services). As there was no detailed list of sheep flocks in Algeria we used instead the list of all municipalities from the studied regions (*n* = 342) as a sampling frame and performed a proportionate random sampling of 56 municipalities with SAS 9.2 (procsurveyselect, strata = region); one flock, supposed to be representative of the municipality, was then selected in each of the 56 municipalities by the private veterinarians working there.

#### Sampling collection

In each flock, at least 10 apparently healthy adult animals, aged between 6 and 24 months, were randomly selected by the veterinarians for blood collection to detect BD antibodies and virus, in addition to some young animals (less than 6 months), depending on the owner’s cooperation, which were sampled for virus detection only. In total, 689 animals were sampled, 576 adults (serum and plasma), and 113 lambs (plasma only). The distribution and number of samples taken in each department are given in Table 1. Each sample (5 ml) was taken from the jugular vein using a vacutainer EDTA tube to collect plasma and a simple vacutainer tube to collect serum. Sera and plasmas were separated from the clotted blood by centrifugation at 1500 g for 15 min, aliquoted into sterile eppendorf tubes of 1 ml. The storage was realized in − 80 °C freezer. Each specimen was marked with a code comprising an individual sample number with the flock identity.

### Survey

A questionnaire was conducted and discussed directly with the sheep owners, during the same visit as the blood sampling, to provide information concerning flocks’ characteristics and epidemiological data: flock size and composition, animal movements and contacts, reproductive management, sanitary situation (abortion, other diseases, and vaccination). All answers were recorded on paper and later on registered in an Excel data base. The number and proportion of flocks according to the main investigated characteristics and management practices are summarized in Table 5.

**Table 5:** Distribution of the investigated flocks (*n* = 56) according to the main investigated characteristics (number and percentage)

Parameter	Category	N Flocks (%)
Climate	Arid	46 (82%)
	Mediterranean	10 (18%)
Landscape	Mountain	7 (12.5%)
	Plateau	49 (87.5%)
Breed	El Hamra + other	27 (48%)
	OuledDjellal + other	22 (39%)
	Local breed	2 (4%)
	Rimbi	5 (9%)
Herd size	< 50	8 (14%)
	51–100	19 (34%)
	101–200	20 (36%)
	> 200	9 (16%)
Management	Sedentary	24 (43%)
	Transhumant	32 (57%)
Herd species	Mixed (sheep/goat/cattle)	40 (71%)
	Ovine only	16 (29%)

### Laboratory testing

#### ELISA for the detection of antibodies to ruminant pestivirus (ab enzyme linked Immuno sorbent assay)

The specific anti-pestivirus antibodies were measured in 576 sera from adult sheep using a commercially available ELISA kit (SERELISA® BVD NS2–3 (p80) Ab Mono Blocking, Synbiotics (Zoetis) according to the manufacturer’s instructions. This kit allows the detection of anti-BVDV and anti-BDV antibodies in ruminants. Optical density (OD) was measured in bichromatism at 450 and 630 nm. Results are expressed as competition percentage resulting from the difference of OD between the negative control and the sample reported to the difference of OD between the negative control and the positive control. According to the manufacturer’s instructions, a sample was considered positive if the competition percentage was superior or equal to 40%, negative below 20% and doubtful between 20 and 40%.

#### Virus neutralization test

Among the 576 sera tested in Ab ELISA, 197 samples (including positive, doubtful and negative samples in Ab ELISA) were tested in parallel for the presence of neutralizing antibodies against BVDV-1, BVDV-2 and BDV using two strains of BVDV (BVDV-1 strain NADL [39], BVDV-2 strain 3534 [40] and the BDV strain AV [41]. The samples were inactivated at 56 °C for 30 min before testing. The inactivated sera were then diluted in minimum essential medium (MEM) in a two two-fold dilution series starting from 1:5 dilution for BVDV-1 and BVDV-2 and from 1:2 for BDV. A fixed virus dose containing 100 TCID50/50 μl (between 30 and 300 TCID50) was incubated for 2 h at 37 °C with each dilution in an antibiotic enriched growth medium (i.e. penicillin, gentamicin and amphotericin B). MDBK cells (ATCC Number CCL-22) (3x10E7 cells/100 μl) were added and the cultures were grown for 72H at 37 °C in a CO2 incubator. All sera were tested in duplicate. Viruses were titrated in all assays. After incubation the cell cultures were evaluated directly for cytopathogenic effects by optical microscopy (BVDV-1) or after immunolabelling with an anti-pestivirus polyclonal serum (BVDV-2 and BDV). The virus neutralizing titers were calculated according to the Reed-Muench method [42]. Titers were expressed as the reciprocal of the highest serum dilution yielding virus growth neutralization and considered as positive for BVDV and for BDV when greater than or equal to 1/10 or 1/8 respectively.

#### ELISA for the detection of ruminant pestivirus antigens (ag enzyme linked Immuno sorbent assay)

Plasma collected from the 689 blood samples were tested for the presence of pestivirus antigen using the SERELISA® Kit BVD NS2–3 (p80) Ag Indirect Mono, Synbiotics (Zoetis). This kit allows the detection of BVDV and BDV antigens in individual samples from PI animals, using a monocupule indirect immuno-enzymatic technique for antigen detection (non-structural protein NS2–3 (p80)/125 common to all strains of BVD and BD viruses). OD was measured in bichromatism at 450 and 630 nm. Results are expressed as an index = 0.5 x OD sample – OD Positive control (P). Any plasma sample having an index ≥ (0.15 x OD P) was considered positive. Any plasma sample with an index < (0.3 x OD P) was considered negative. Any plasma sample with an index between (0.15 x OD P) and (0.3 x OD P) was considered doubtful according to the manufacturer’s instructions.

#### RT-PCR (real time-polymerase chain reaction)

RT-PCR were performed on pools of plasma samples from 10 different animals which were constituted by mixing together 100 μl of each individual sample. RNA (Ribonucleic acid) was extracted from each pool using a volume of 100 μl. Extraction was performed with the QIAamp Viral RNA Mini Kit (Qiagen GmbH, Hilden, Germany) according to the manufacturer’s instructions. Four μl of the total extracted RNA were used for the reverse transcription in the presence of hexanucleotides [43]. For real-time PCR amplification, 5 μl of the resulting cDNAs were included in the reaction mix. The primers (F2: CTCGAGATGCCATGTGGAC and PESTR: CTCCATGTGCCATGTACAGCA) and TaqMan probes used in this study targeted the 5′UTR conserved regions of BD and BVDV genotype 1 (probe BVDV-1: ^5’^ FAM-CAGCCTGATAGGGTGCTGCAGAGGC-TAMRA ^3′^) and of BVDV genotype 2 (probe BVDV-2: ^5’^ VIC-CACAGCCTGATAGGGTGTAGCAGAGACCTG-TAMRA ^3′^) [44]. PCR reaction was run in 25 μl containing 2X FastStart DNA Taqman probe Master Mix (LifeScience), 450 nM of both primers and 50 nM of both fluorescent probes. The PCR conditions were as followed: 10 min at 95 °C and 45 cycles with 15 s at 95 °C and 45 s at 60 °C. Fluorescent measurements were carried out during the elongation step.

### Statistical analysis

#### Seroprevalence estimation

In order to take into account the clustering effect (~ 10 animals were sampled in each flock), the within-flock seroprevalence was estimated at the overall level and at department level with a generalized estimating equation model (GEE) using SAS 9.2 software (“proc genmod”). In this model, the flock was taken as repeated subject, the department as an independent variable and the prevalence was estimated as the predictive probability to be seropositive; an exchangeable correlation matrix was assumed. Doubtful Ab-ELISA results, based on the cut-off recommended by the kit manufacturer, were considered in our analysis as positive, given that we found that most samples that were doubtful in Ab-ELISA were positive with the VNT (see results section). These prevalence rates were apparent prevalence (Pa), not taking into account the sensitivity (Se) and specificity (Sp) of the Ab-ELISA. The overall true seroprevalence (Pt) was then calculated taking into account the Se and Sp that we estimated relatively to the VNT (see results section). The true overall prevalence and 95% CI (Rogan and Gladen method) were calculated using the on-line epidemiological calculator EpiTools (Estimated true prevalence and predictive values from survey testing, [45]).

#### Comparison between ab-ELISA and VNT results

A comparative study was performed on part of the serum samples (196/576) to estimate the performances of the Ab-ELISA compared to the VNT for BDV, BVDV-1 and BVDV-2. These samples included negative, doubtful and positive sera in Ab ELISA originating from 20 different flocks and 8 different departments. The relative sensitivity and specificity of the ELISA were calculated as the number of positive or negative samples in ELISA divided by the number of positive or negative samples in VNT, respectively. The Cohen’s kappa coefficient test was used to measure the agreement between Ab-ELISA and the VNT and was calculated using the on-line epidemiological calculator EpiTools [45]. Doubtful Ab-ELISA results were considered as positive in the calculations mentioned above.

#### Descriptive statistics and risk factors

Descriptive statistics were performed to establish the proportion of flocks according to the different characteristics studied through the survey and the corresponding proportion of seropositive animals. Doubtful Ab-ELISA results were considered as positive as explained above. The following parameters, considered as potential risk factors, were compared in terms of seroprevalence: climate (arid vs/ Mediterranean), landscape (mountain vs/plateau) flock management (sedentary vs/transhumant), flock size (< 100 vs > 100), flock composition (sheep/cattle/goats), sheep breed, purchase of breeding females (yes/no), purchase of fattening lambs (yes/no), origin of breeding rams (external vs/own ram), contacts with other flocks at pasture, contacts with wild animals, number of abortions, clinical diseases, vaccination. A chi-squared test was used to detect significant differences in seroprevalence for the studied characteristics; a probability of less than 5% was considered as statistically significant. The odds ratio (OR) and chi-square were calculated with the software XLSTAT version 2014 to quantify the association between positive Ab-ELISA and the identified risk factors. The 95% CI were calculated using the Miettinen method.

## Additional file

Additional file 1: List of all samples with their respective Ab-ELISA and VNT results. (XLSX 44 kb)

## Abbreviations

Ab-ELISA: Antibody enzyme-linked immunosorbent assay
Ag-ELISA: Antigen enzyme-linked immunosorbent assay
BD: Border Disease
BDV: Border Disease Virus
BVDV: Bovine Viral Diarrhea virus
C.I.: Confidence interval
CSFV: Classical Swine Fever virus
GEE: Generalized estimating equation model
ICC: Intra-class correlation
MDBK cells: Madin-Darby Bovine Kidney cells
MEM: Minimum essential medium
OD: Optical Density
OIE: World Organisation for Animal Health
OR: Odds ratio
P: Seroprevalence
Pa: Apparent seroprevalence
PI: Persistently Infected
Pt: True seroprevalence
RNA: Ribonucleic acid
RT-PCR: Real Time-polymerase chain reaction
Se: Sensitivity
Sp: specificity
TCID: Tissue culture infectious dose 50%
VNT: Virus neutralization test
